# 

**Published:** 1877-10-20

**Authors:** 


					﻿VOL. VII.	OCTOBER 20, 1877	_	No. 10.
THE
SOUTHERN MEDICAL RECORD.
—y- ■	'	... , ; r
A MONTHLY JOURNAL OF PRACTICAL MEDICINE.
“ QUICQUIDPR&XJIPIES ESTO BREVIS. ”
ffl
§
j'p
• A
P
-•»
Ip
o
;o
A
(g
• Ph
■	iP
P
O
QQ
1 5
WK
3
! Q
W.
. A
EH
H
: a
ri p
■	®
P
A
-■ P
: p
1 00
tP-
■-! A,
: cu
II
02 L
w
O I
» t
h.:
Q •
Q .
g .
g
’ d
1 a
■ w.
a.
►
i—f ■
O .'
a .
.00 ;
> -
a ■
w i
> i
1
I—I
A
s
g:
I
00 ;
£
M
o
I—I 1
i
« I
d I
ii
H • I
JAS. P. HARRISON & CO., Printers.
ATLANTA, GEORGIA.
TERMS: $2.00 per Anuum in Advance.—Club Rates, 5 Copies for $8.00.
				

